# Chromosome-scale genome assembly of an acidophilic microalga *Tetratostichococcus* sp. P1 isolated from a tropical peatland in Malaysia

**DOI:** 10.1128/MRA.00816-23

**Published:** 2024-01-05

**Authors:** Yoshiaki Maeda, Toshiki Hirakawa, Shigekatsu Suzuki, Noraiza Suhaimi, Nurul Syahirah Shamsol Anuar, Kohei Yoneda, Masaki Shintani, Kenshi Suzuki, Naoki Sunagawa, Masanobu Kawachi, Iwane Suzuki, Hirofumi Hara

**Affiliations:** 1 Institute of Life and Environmental Sciences, University of Tsukuba, Tsukuba, Japan; 2 Graduate School of Life and Environmental Sciences, University of Tsukuba, Tsukuba, Japan; 3 Biodiversity Division, National Institute for Environmental Studies, Tsukuba, Japan; 4 Department of Biotechnology, Graduate School of Agricultural and Life Sciences, The University of Tokyo, Tokyo, Japan; 5 Department of Engineering, Graduate School of Integrated Science and Technology, Shizuoka University, Shizuoka, Japan; 6 Department of Biomaterial Sciences, Graduate School of Agricultural and Life Sciences, The University of Tokyo, Tokyo, Japan; 7 School of Integrative and Global Majors, University of Tsukuba, Tsukuba, Japan; 8 Department of Chemical and Environmental Engineering, Malaysia-Japan International Institute of Technology, Universiti Teknologi Malaysia, Kuala Lumpur, Malaysia; University of Maryland School of Medicine, Baltimore, Maryland, USA

**Keywords:** acidophilic microalga, tropical peatland, *Tetratostichococcus*

## Abstract

*Tetratostichococcus* sp. P1 shows an acidophilic phenotype which could allow mass-scale monoculture of this green microalga without severe contamination by environmental microorganisms. In this study, we report a chromosome-scale genome assembly for *Tetratostichococcus* sp. P1.

## ANNOUNCEMENT

*Tetratostichococcus* sp. P1 was isolated in 2016 from the tropical peatland in Malaysia (Raja Musa Peat Swamp Forest Reserve, Hulu Selangor, 0″N 101°26′31, 3°27′58, 5) and has unique characteristics of fatty acid production and adaptation of low pH conditions. To elucidate the molecular mechanism of these unique phenotypes, the genome of this strain was previously sequenced using Ion S5 XL (1). However, the draft genome information is highly fragmented into 15,474 contigs (N_50_: ~6 kbp), and approximately 95% of these contigs are shorter than 10 kbp, and 33% are <1 kbp. Low continuity of the draft genome information could negatively affect further investigation including transcriptomics to study the acidophilic phenotype. Therefore, we performed the re-sequencing of the genome of *Tetratostichococcus* sp. P1 using long-read PacBio technology.

*Tetratostichococcus* sp. P1 was cultivated in 500 mL of the modified BG-11 (1) at 25°C with aeration (2% CO_2_) under continuous illumination (70 µmol photons/m^2^/s) for 7 days. DNA was extracted using Genomic-tip 20/G (QIAGEN N.V., Hilden, Germany), followed by fragmentation into approximately 10–20 kbp using g-TUBE (Covaris LLC., Woburn, MA, USA). SMRTbell Express Template Prep Kit 2.0 (PacBio, Menlo Park, CA, USA) was used for library preparation. Sequencing was performed using Sequel IIe with Binding Kit 2.2 (PacBio). SMRT Link (ver. 12.0.0.177059) was used to remove the overhang adopter from the reads. Subsequently, high-fidelity reads (>Q20) longer than 1 kbp were selected using Filtlong ver.0.2.1 (2), and assembled using Hifiasm ver. 0.19.3-r572 (3). Gene prediction and functional annotation were performed using Funannotate pipeline v.1.8.15 (4), of which augustus was replaced by BRAKER v.3.0.3 (5) with the option of --augustus_gff. Default parameters were used except where otherwise noted.

Sequencing using PacBio technology generated 119,625 reads (~1.3 Gbp, read N_50_: ~13 kbp). The genome assembly was approximately 49.4 Mbp contained in 22 contigs (N_50_: 4.1 Mbp, Table 1), namely 17, 3, and 2 contigs corresponding to the genome of the nucleus, chloroplast, and mitochondrion, respectively. The total size and N_50_ value of the contigs of the nucleic genome are approximately 48.9 and 4.1 Mbp, respectively. These results indicate obvious improvement in genome assembly as compared to the previous report (Table 1) (1). Among 17 contigs of the nucleic genome, 9 have telomeric repeats (5′-CCCTAAA-3′) at both ends, and 7 have them at either end. These data suggest that the present genome assembly reached the chromosome level. One short contig (12,266 bp) containing a small subunit ribosomal RNA gene (Accession number: MT053478.1) does not have the telomeric repeats, and this could be caused by arresting the assembling process by rRNA gene repeats. The GC content of the nuclear genome is 58.84%. As a result of gene prediction and annotation, 9,189 genes were predicted. The reduction of the predicted genes in the present analysis might be caused by the overestimation of the number of genes in the highly fragmented assembly in the previous study (Table 1). The re-sequenced genome assembly will provide insights into the molecular mechanism of unique phenotypes of *Tetratostichococcus* sp. P1.

**TABLE 1:** Comparison of genome assembly of *Tetratostichococcus* sp. P1

Technology	No. of contig	Total size of contigs (Mbp)	N_50_ value of contigs (kbp)	No. of predicted genes	Reference
Ion S5 XL (Ion Torrent)	15,474	52.0	6	17,582	(1)
Sequel Iie (PacBio)	22	49.4	4,066	9,189	This study

## Data Availability

Sequences were deposited in DDBJ under BioProject accession number PRJDB16267 and BioSample accession number SAMD00632675. PacBio reads are available under DRA Run accession number DRR495341. Accession numbers of 22 contig sequences are BTPI01000001-BTPI01000022.
